# Hypoglycemic Effect of Two Mexican Medicinal Plants

**DOI:** 10.3390/plants10102060

**Published:** 2021-09-29

**Authors:** Adolfo Andrade-Cetto, Fernanda Espinoza-Hernández, Gerardo Mata-Torres, Sonia Escandón-Rivera

**Affiliations:** Laboratory of Ethnopharmacology, School of Sciences, National Autonomous University of Mexico, Mexico City 04510, Mexico; f.artemisa.ehdz@ciencias.unam.mx (F.E.-H.); gerardom.torres@ciencias.unam.mx (G.M.-T.); soniaer@ciencias.unam.mx (S.E.-R.)

**Keywords:** traditional medicine, hypoglycemic plants, *Eryngium longifolium*, *Alsophila firma*, glucose 6-phosphatase, fructose 1,6-bisphosphatase

## Abstract

Type 2 diabetes is a worldwide prevalent disease that is due to a progressive loss of adequate β-cell insulin secretion, frequently against a background of insulin resistance. In Mexican traditional medicine, the therapeutic use of hypoglycemic plants to control the disease is a common practice among type 2 diabetic patients. In the present work, we examined the traditional use of the aerial parts of *Eryngium longifolium* and the rhizome of *Alsophila firma*, consumed by people use over the day (in fasting state) to control their blood glucose levels, therefore, we aimed to assess the acute hypoglycemic effect of both plants. First, basic phytochemical profiles of both plants were determined and, subsequently, acute toxicity tests were carried out. Then, in vivo hypoglycemic tests were performed in streptozotocin-nicotinamide (STZ-NA) induced hyperglycemic Wistar rats and finally the effect of the plants on three enzymes involved in glucose metabolism was assayed in vitro. Through HPLC-DAD chromatography, caffeic acid, chlorogenic acid, rosmarinic acid, isoflavones, and glycosylated flavonoids were identified in *E. longifolium*, while the possible presence of flavanones or dihydroflavonols was reported in *A. firma*. Both plants exhibited a statistically significant hypoglycemic effect, without a dose-dependent effect. Furthermore, they inhibited glucose 6-phosphatase and fructose 1,6-bisphosphatase in in vitro assays, which could be associated with the hypoglycemic effect in vivo. Thus, this study confirmed for the first time the traditional use of the aerial part of *E. longifolium* and the rhizome of *A. firma* as hypoglycemic agents in a hyperglycemic animal model. In addition, it was concluded that their ability to regulate hyperglycemia could involve the inhibition of hepatic glucose output, which mainly controls glucose levels in the fasting state.

## 1. Introduction

Traditional medicine (TM), as defined by the Word Health Organization (WHO), includes medication therapies that involve the use of herbal medicines, animal parts and/or minerals; the term is used to refer to various forms of indigenous medicine [1]. In the case of Mexico, TM has not been incorporated into the national health care system and conversely the official system is mainly based on allopathic medicine. However, many people still rely in the use of medicinal plants to treat health problems, thus the use of plants in diseases like type 2 diabetes (T2D) is a common practice [2,3]. The American Diabetes Association (ADA) states that T2D is due to a progressive loss of adequate β-cell insulin secretion, frequently on the background of insulin resistance, which manifests clinically as hyperglycemia. Once hyperglycemia occurs, patients are at risk of developing chronic complications, such as microvascular and macrovascular diseases, and acute coronary syndrome [4]. T2D accounts for 90–95% of all diabetes, the last report of the International Diabetes Federation (IDF) ranks Mexico as sixth in the world, with 12.8 million T2D patients [5]. In T2D patients, two sources that contribute to raise blood glucose levels are food ingestion, which increases glucose levels in postprandial state, and the liver glucose output, which promotes high glucose levels in the fasting state. In these metabolic processes, the intestinal α-glucosidases, and the hepatic glucose 6-phosphatase (G6Pase) and fructose 1,6-bisphosphatase (FBPase) enzymes play an important role [6,7].

Mexico stands out among the mega-diverse countries, being the fourth nation in terms of species richness with the presence of all the climates of the planet [8]. It is calculated that more than 6000 plants are used for medicinal purposes. Moreover, around 30 million people live in rural areas, about 12 million belong to one of the 86 ethnolinguistic indigenous groups that make up about 10% of the population [9], and 80% of the population are poor or vulnerable (due to social deprivation or low income) [10]. These aspects, combined with the richness in medicinal plants and the large number of ethnic groups, makes Mexico an important source of traditionally used medicinal plants. Considering the high amount of T2D patients, the perfect combination is presented for the exploration and study of traditionally used hypoglycemic plants. Hence, two plants used in the municipalities of Tlanchinol and Huejutla de Reyes (Hidalgo), located in central Mexico, were selected for this study. Both plants were already reported by our group as hypoglycemic species based on previous ethnopharmacological field work [3].

**Figure 1:**
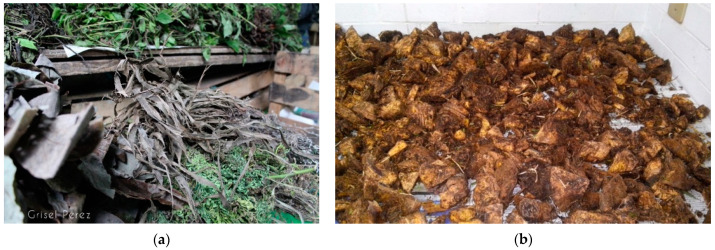
Photographs of the study plants ready for consumption: (**a**) Dried aerial part of *E. longifolium*; (**b**) Rhizome of *A. firma*.

*Eryngium longifolium* Cav. (Apiaceae) (Figure 1a), which is native and endemic to Mexico, is a herb of up to 3 m in height with simple, lobed, or spiny-toothed to linear leaves, varied venation, flower in bracted heads, and globose or ovoid fruit [11]. In addition to being used in the treatment of diabetes, it has also been reported as a diuretic, emmenagogue, and alexiteric [12]. More information of the plant can be found at https://enciclovida.mx/especies/171163-eryngium-longifolium (access on 25 September 2021).

On the other hand, *Alsophila firma* (Baker) D.S.Conant (Cyatheaceae) (Figure 1b) is an arborescent fern with spiny stems up to 10 m high, yellowish-brown in color, with a blade up to 3.3 × 1.5 m, 2-pinnate-pinnatifid. It can be found in temperate and humid forests. It sometimes presents adventitious buds, veins without trichomes or with few irregular dark squamules at their base abaxially; globose to subglobose indusian, with or without an umbo, very delicate, fleeting at maturity, usually glabrous, diaphanous to yellowish-brown [11]. Scales from the fronds are used to stop hemorrhages [13]. More information about the plant can be found at https://enciclovida.mx/especies/151254-alsophila-firma (access on 25 September 2021).

Until now, no pharmacological studies about these species are reported in the international literature. Therefore, the present work aims to contribute to the overall knowledge of *E. longifolium* and *A. firma*, two plants therapeutically used by T2D patients as hypoglycemics. The acute hypoglycemic effect of the traditionally used aqueous extract and the ethanol-water (EtOH) extract as well as their possible dose-dependent effect were tested in hyperglycemic rats. Additionally, the effect of the extracts on two of the main enzymes involved in the gluconeogenesis pathway (G6Pase and FBPase) as well as the main enzyme involved in the intestinal carbohydrate breakdown (α-glucosidase) was tested. Also, acute toxicity tests were carried out and basic phytochemical profiles of the extracts that had a higher biological activity in the in vivo tests were provided.

## 2. Results

### 2.1. Ethnobotany

According to the information obtained from the specialists, sellers, and diabetic patients, the traditional use of *E. longifolium* by the patients from the town of Tlanchinol and patients who visit the Huejutla de Reyes market to control their blood glucose levels was confirmed. Both locations are in Hidalgo, Mexico. Particularly, the traditional healer Isabel Escalante recommended the use of the plant *E. longifolium*, known by its Spanish name “piñuela”. It is prepared as an infusion, in which 20 g of the dry plant (aerial part) are placed in 500 mL of boiling water. When the infusion reaches room temperature, it is consumed over the day as so-called “agua de uso”.

On the other hand, the specialist Guadalupe Vite specifically recommends *A. firma* for controlling blood glucose levels of diabetics in Tlanchinol, where the plant is known by its Nahuatl name “peshma”. The patients also prepare an infusion with 20 g of the dried rhizome (powder) in 500 mL of boiling water. After filtration, it is also consumed as “agua de uso”.

It is important to note that since both plants are consumed as “agua de uso”, they are mainly consumed in the fasting state, namely they are drunk throughout the day instead of normal water and not with the meals.

### 2.2. Chromatographic Profiles

**Figure 2:**
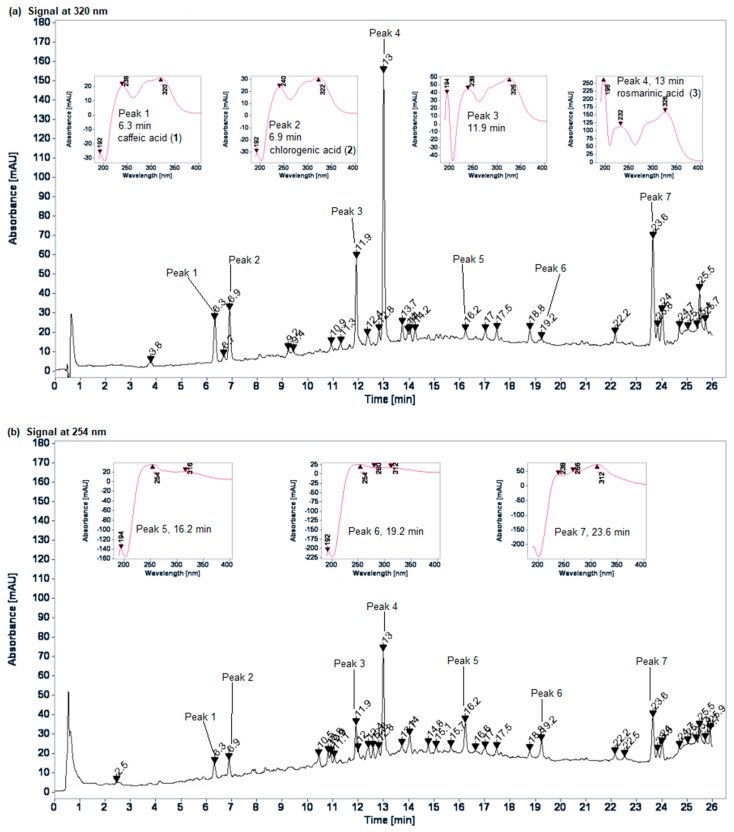
HPLC fingerprint profile of the *E. longifolium* EtOH extract: (**a**) 320 nm; (**b**) 254 nm.

According to the in vivo outcomes, the *E. longifolium* EtOH extract and the *A. firma* aqueous extract presented the best hypoglycemic activity. The HPLC fingerprint profile of the *E. longifolium* EtOH extract shows the most important signals at 254 and 320 nm (Figure 2). Seven major peaks (at 6.3, 6.9, 11.9, 13.0, 16.2, 19.2 and 23.6 min from 1 to 7, respectively) and several minor peaks were observed. The UV spectra of 1, 2 and 4 revealed characteristic signs of known phenolic acids which were identified by comparing the retention time and the UV spectrum with commercial standards (caffeic acid, **1**; chlorogenic acid, **2**; rosmarinic acid, **3**). Peaks 3 and 4 have the same spectral characteristics, while peaks 5 and 6 shows UV spectral typical signs of isoflavones, with an intense Band II absorption (λ_max_ 254 nm in both cases) and Band I with a very low intensity (λ_max_ 316 and 312 nm, respectively) [14] and, peak 7 shows signs of glycosylated flavonoids as previously reported in other *Eryngium* species [15,16].

**Figure 3:**
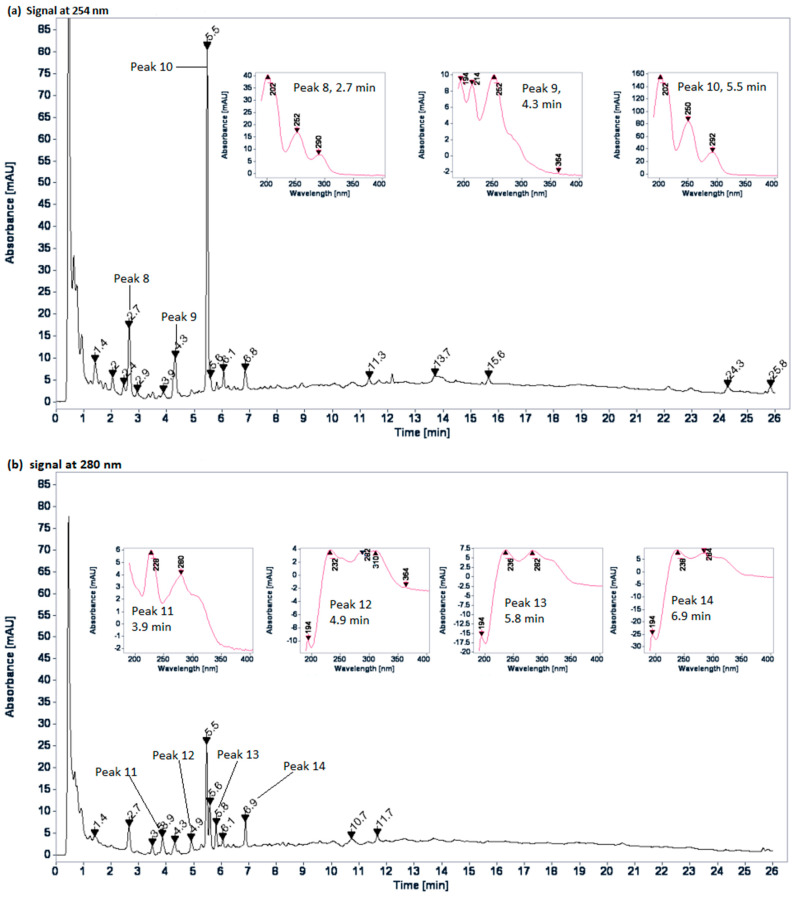
HPLC fingerprint profile of the *A. firma* aqueous extract: (**a**) 254 nm; (**b**) 280 nm.

The HPLC fingerprint profile of the *A. firma* aqueous extract shows the most important signals at 254 and 280 nm (Figure 3), i.e., peaks with characteristic phenolic UV spectra probably due to flavanones or dihydroflavonols (peaks 8 to 14). Peak 10 in particular has a UV spectrum with this property [14].

### 2.3. Acute Oral Toxicity Tests

To assess a possible acute toxic effect of the tested extracts, acute toxicity tests were carried out according to the guidelines for the Testing of Chemicals of the Organisation for Economic Co-operation and Development (OECD) [17]. The results showed no physical or behavioral abnormalities after oral administration of the maximum dose of 2000 mg/kg body weight (b.w.) of each extract. Also, no deaths were reported. Therefore, traditional doses, and elevated-traditional doses used for this study are within the safe range, indicating that the acute exposure of these plants does not generate toxic effects and the LD_50_ is greater than the maximum dose used.

### 2.4. Hypoglycemic Effect of Plant Extracts

As shown in Table 1, both normoglycemic control and negative hyperglycemic control exhibited stable and unchanged blood glucose values throughout the acute test, indicating that physiological solution (vehicle) administration did not alter this parameter over time despite the physiological condition of the animals.

Comparing both groups, the hyperglycemic control shown statistical differences against normoglycemic control in all times through the 3 h. The pancreatic damage caused by STZ administration was such that blood glucose levels rose significantly around 190 mg/dL in the hyperglycemic control compared with those observed in the normoglycemic control. However, the oral administration of the hypoglycemic agent glibenclamide turned out in a significant decrease in these levels from the first hour, reaching normoglycemic levels at the second hour of the test. In addition, glibenclamide shown statistical differences against hyperglycemic control from 60 to 180 min. These outcomes support the idea that this hyperglycemic model is adequate to assess the hypoglycemic effect of mixtures or isolated compounds since it can respond to hypoglycemic drugs, such as glibenclamide, despite its reduced insulin levels.

**Figure 4:**
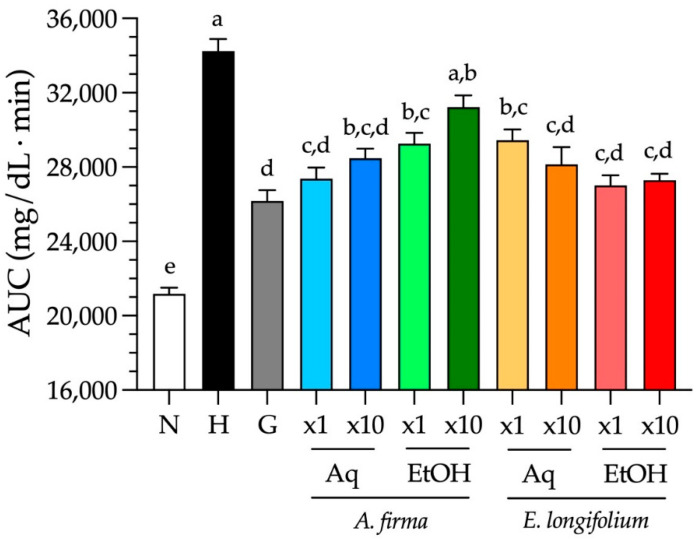
Blood glucose AUC from 3-h acute tests (mean ± SEM, *n* = 9). Different letters over bars indicate statistically significant difference among groups at *p* < 0.05 (a > b > c > d > e). N–normoglycemic control; H–hyperglycemic control; G–hyperglycemic + glibenclamide control; x1–traditional dose; x10–traditional high dose; Aq–aqueous extract; EtOH–ethanol-water extract.

Regarding plant extracts, all of them could effectively decrease blood glucose levels though none displayed a dose-dependent effect, namely no statistical differences between the doses of each type of extract were observed. The aqueous extract of *A. firma* at its traditional dose shown a better glycemic control than its EtOH one at both doses, while *E. longifolium* reached normoglycemic levels at the end of the experiment with its two types of extracts, mainly with the EtOH one at its traditional dose. Particularly, although the EtOH extract of *A. firma* high dose could improve hyperglycemia by significantly lowering around 50 mg/dL of blood glucose, it controlled overall glycemia in a modest way as compared with the other extracts after 3 h of treatment, as shown in Figure 4. Taken together, these results support the traditional use of *A. firma* and *E. longifolium* for the treatment of T2D. It is suggested that future experiments on their mechanism of action be investigated using the traditional doses of the aqueous extract of *A. firma*, and the EtOH extract of *E. longifolium* since both were as effective as glibenclamide in lowering glucose levels.

### 2.5. Inhibition of Key Hyperglycemia-Related Enzymes

**Figure 5:**
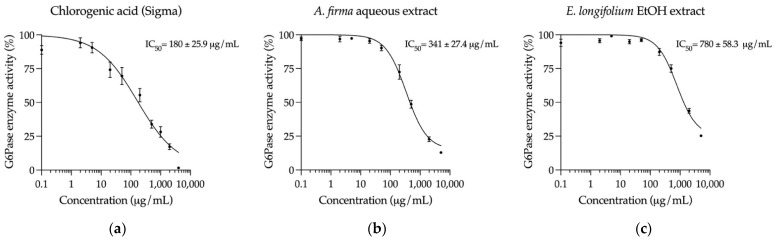
Concentration-response inhibition curves on G6Pase (mean ± SEM, *n* = 6): (**a**) Curve of chlorogenic acid (control); (**b**) Curve of *A. firma* aqueous extract; (**c**) Curve of *E. longifolium* EtOH extract.

Impaired hepatic glucose production, specifically gluconeogenesis, is the main source of fasting hyperglycemia and is one of the contributors of postprandial hyperglycemia in diabetic patients [6]. Therefore, the *A. firma* aqueous extract and the *E. longifolium* EtOH extract were evaluated on two of the principal enzymes involved in this pathway: G6Pase and FBPase. Regarding G6Pase, each plant extract effectively inhibited its activity. As shown in Figure 5, even though the control chlorogenic acid had the strongest effect on this enzyme, both extracts could decrease the enzyme activity in the same order of magnitude. The *A. firma* aqueous extract diminished G6Pase activity by 87% at the highest concentration (5000 μg/mL), while *E. longifolium* EtOH extract by 75%.

**Figure 6:**
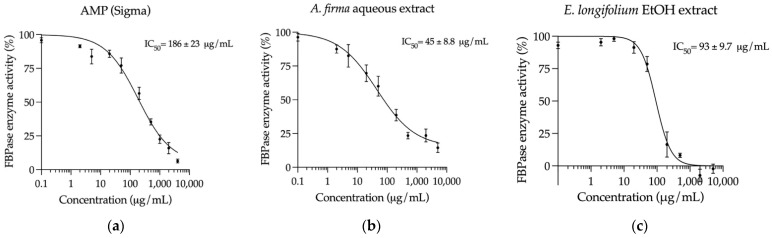
Concentration-response inhibition curves on FBPase (mean ± SEM, *n* = 6): (**a**) Curve of AMP (control); (**b**) Curve of *A. firma* aqueous extract; (**c**) Curve of *E. longifolium* EtOH extract.

In contrast to what was observed for G6Pase activity, both extracts exhibited a stronger inhibitory effect on FBPase activity than the control adenosine 5′-monophosphate (AMP), as shown in Figure 6. Interestingly, the *E. longifolium* EtOH extract inhibited the enzyme activity by 100% at the highest concentration (5000 μg/mL), being more potent than AMP (92%), while the *A. firma* aqueous extract reduced the FBPase activity by 86%.

**Figure 7:**
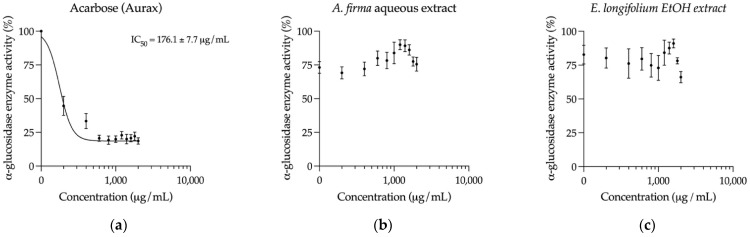
Concentration-response inhibition curves on intestinal α-glucosidase enzymes (mean ± SEM, *n* = 6): (**a**) Curve of acarbose (control); (**b**) Curve of *A. firma* aqueous extract; (**c**) Curve of *E. longifolium* EtOH extract.

Finally, the effect of the *A. firma* aqueous extract and the *E. longifolium* EtOH extract on intestinal α-glucosidase enzymes was assessed. Among other mechanisms, these enzymes are responsible for the postprandial hyperglycemia in diabetic patients since they hydrolyze diet’s oligosaccharides for their subsequent absorption into simple monomers after their consumption [7]. As shown in Figure 7, neither the *A. firma* aqueous extract nor the *E. longifolium* EtOH extract exerted a substantial inhibition on intestinal α-glucosidase enzymes, suggesting that these plants are not capable of producing a significant reduction of postprandial hyperglycemia through the inhibition of carbohydrate breakdown.

## 3. Discussion

T2D is an emerging disease in Mexico. In the 19th century, only one death per year was attributable to diabetes. Since 1950, there has been an increase in the mortality rates of this disease, which went from 0.2 to 31.7 per 100,000 inhabitants in 1990. In 2019, the prevalence was 13.5, placing the country in sixth place in the world [5,18]. Moreover, the use of medicinal plants to treat any disease is part of the Mexican idiosyncrasy and a disease such as T2D, with high prevalence, is no exception. As a result of ethnopharmacological research, we found that the Mexican population (herbal specialists, plant sellers, and T2D patients) are looking for new medicinal plants to counteract the high glucose levels produced by the disease. 

This phenomenon is observed in other Latin American countries. For instance, it is documented that 91% of the Cakchiquels, a Guatemalan ethnicity, with T2D use medicinal plants in addition to their medical prescription [19]. The local usage of medicinal plants is related to plant diversity and traditional knowledge [20], therefore, the richness of plant species in America gives the opportunity to use a great variety of plants for medicinal purposes.

In 2005, Barbosa-Filho et al. reported 224 hypoglycemic plants supported by scientific studies from North, Central, and South American countries [21]. However, only in that year, 306 Mexican species popularly used as hypoglycemic agents were documented [22], and by 2020, it was estimated the use of about 800 plants in Mexico for treating T2D [23]. These growing numbers indicate that although traditionally used plants continue to be identified through ethnopharmacological approaches, works that support their hypoglycemic effect, their phytochemical composition, and their associated mechanisms of action are still lacking.

In Mexico, T2D affects nearly 13 million people [5], who usually treat it with hypoglycemic plants. Particularly, we detected the use of the aerial part of *E. longifolium* and the rhizome of *A. firma* to control the disease among healers and T2D patients in the municipalities of Tlanchinol and Huejutla, in the state of Hidalgo. For this purpose, an infusion is prepared and consumed over the day, mainly in the postabsorptive state, where glucose production by the liver plays a crucial role in maintaining blood glucose levels [24]. Our findings suggest that the hypoglycemic effect exerted in vivo by *E. longifolium* and *A. firma* could be correlated with the regulation of hepatic glucose output since both plants were able to inhibit two of the key enzymes that participate in gluconeogenesis: G6Pase and FBPase.

The sustained hypoglycemic effect of the EtOH extract of *E. longifolium* and the aqueous extract of *A. firma* is comparable to that observed for glibenclamide. Likewise, other species used in Central and South America have proved to decrease blood glucose levels in the same way as this hypoglycemic drug, such as the ethanolic leaf extract of *Phyllanthus acidus* (L.) Skeels (Phyllanthaceae) [25], the methanolic extract of *Eclipta prostrata* (L.) L. (Compositae) [26], the ethanolic bark extract of *Croton guatemalensis* Lotsy (Euphorbiaceae), and the ethanolic leaf extract of *Solanum americanum* Mill. (Solanaceae) [27]. Furthermore, other South American species like *Clusia latipes* Planch. & Triana (Clusiaceae) [28] and *Terminalia phaeocarpa* Eichler (Combretaceae) [29] shown to possess inhibitory activity on α-glucosidases, which was reported as not significant in the Mexican species in this study. However, even though both plants presented a modest inhibition of α-glucosidases (around 25%), this mechanism could contribute to the hypoglycemic effect in the postprandial state in a synergistic way, namely the possible reduction of postprandial hyperglycemia by these plants could be mainly due to other mechanisms, such as insulin secretion, improvement of insulin function, and blocking of intestinal absorption, rather than inhibition of carbohydrate breakdown. In this regard, it is necessary to perform carbohydrate load curves in further works with the aim to prove the anti-hyperglycemic effect of these medicinal plants.

Moreover, we confirmed that the STZ-NA hyperglycemic model is suitable for testing plant extracts. Although this model does not present all the characteristics of T2D, it exhibits a stable hyperglycemia that can be controlled with a hypoglycemic agent, such as a sulfonylurea, due to the responsiveness to insulin secretagogues [30]. Nevertheless, different type of models could be considered to assess other therapeutic aspects of these plants, such as the improvement of insulin resistance.

As can be observed in the phytochemical profile of *E. longifolium* EtOH extract (Figure 1), phenolic acids and flavonoids are the predominant components, which agrees with the chemical profiles previously reported in other *Eryngium* species [31]. Rosmarinic acid (**4**) was identified by comparing it with a commercial standard and it was noted as the most abundant in the extract. This important antioxidant has also been identified as the most abundant compound in a hydroalcoholic extract of *E. viviparum* [32] and the second one in an aqueous extract of *E. cymosum* [33]. On the other hand, flavonol glycosides have been isolated and identified in several species of *Eryngium* [31], while isoflavones are less common. Ayuso et al. reported, in 2020, the presence of three tectorigenin glycosides for the first time in the genus [32]. However, more chemical analysis is needed to identify this kind of flavonoids in *E. longifolium*.

On the other hand, Figure 2 shown the phytochemical profile of *A. firma* aqueous extract. It can be noted that the compounds have very high polarity and present characteristics of flavonoids that exhibit no conjugation between the A- and B-rings [14]. Moreover, it should be noted that the intensity of the signals obtained in this chromatographic profile are low, which may indicate the presence of one or more abundant compounds that do not absorb UV light. Further phytochemical analyzes are required to identify the major compounds.

According to the phytochemical profiles provided for both plants, most compounds that can be noted are phenolic compounds. In addition to having strong antioxidant properties, these types of phytochemicals are associated to favorable effects on glucose and lipid metabolism [34,35]. In fact, they can be found not only in medicinal plants, but also in functional foods consumed worldwide, having a good impact in human health and disease [36]. The chronic administration of the major compound identified in the *E. longifolium* EtOH extract, rosmarinic acid (**4**), has shown to reduce the activity of G6Pase and FBPase in STZ-hyperglycemic rats fed with a high-fat diet [37], while chlorogenic acid (**2**) is a well-known inhibitor of G6Pase and a weak inhibitor of α-glucosidases [38].

The principal objective of the current study was to evaluate the acute hypoglycemic effect of the traditionally used *E. longifolium* and *A. firma*, but it would be necessary to assess their sustained effect over time and possible chronic toxicological effects to evaluate their safety in further experiments. We did not detect any conservation issues with these plants, and in the future they could be cultivated in their natural habitats to produce standardized hypoglycemic agents. The present work stablishes the bases of the hypoglycemic activity of these two plants used in Mexican traditional medicine.

## 4. Materials and Methods

### 4.1. Ethnobotany

In a previous ethnobotanical work [3], we detected the use of *E. longifolium* and *A. firma* by T2D patients and two herbal specialists in the town of Tlanchinol, Hidalgo. While *E. longifolium* was reported in the original work, *A. firma* was not included in the cited study because at that time only its common name was known, without a correct botanical identification. To corroborate the previous ethnobotanical information in this work, direct interviews were performed to the two herbal specialists from the town of Tlanchinol, five sellers from the Huejutla market, and 10 diabetic patients who visited the specialists or the market. All of them confirmed the use of the plants for the treatment of T2D. For this work, interviews were carried out to corroborate the part used, dose, preparation, and administration of the plants. The interviews were in free format, asking specific questions about the recollection, preparation, used doses, and administration form of the plants. With the help of the specialist Isabel Escalante, *E. longifolium* was purchased in the Huejutla market, and a voucher specimen (IMSS16170) was deposited in the Mexican Institute of Social Security (IMSS) herbarium, Mexico City. The rhizome of *A. firma* was collected near Tlanchinol, in “La Tangente”, with the help of the specialist Guadalupe Vite, and a voucher specimen (Etnof228) was deposited in the UNAM School of Sciences herbarium, (Mexico City, Mexico).

### 4.2. Elaboration of Traditional Extracts and Dose Calculation

Aqueous and EtOH extracts of the plants were prepared. For the aqueous extracts, 20 g of the dry and ground plant material stirred in 500 mL of boiling water for 15 min and then the mixture was filtered and lyophilized. For the EtOH extracts, 20 g were added to 500 mL of an ethanol-water mixture (1:1) and then heated at 40 °C for 4 h; subsequently, it was filtered and subjected to evaporation in a rotary evaporator (Büchi, Flawil, Switzerland); finally, the whole process was repeated once more and at the end both extracts were mixed and lyophilized.

The traditional dose of each extract was calculated based on the 20-g consumption of a plant by a 70-kg person, namely from the initial 20 g, the yield of each extract was obtained and expressed as mg/kg. To assess a possible dose-dependent response, a high dose was calculated by multiplying the traditional dose by 10 for each type of extract (elevated-traditional dose).

### 4.3. Chemicals and Reagents

Nicotinamide (N0636), streptozotocin (S0130), ethylenediaminetetraacetic acid (EDS), 4-(2-hydroxyethyl)piperazine-1-ethanesulfonic acid (H3375), imidazole (I0250), sodium dodecyl sulfate (L3771), ascorbic acid (A7506), malachite green (M6880), Tween^®^ 20 (P1379), Tris-HCl (T3253), MgCl_2_ (M9272), glucose 6-phosphate (G7879), fructose 1,6-bisphosphate (F6803), adenosine 5′-monophosphate (A2252), 4-nitrophenyl α-D-glucopyranoside (N1377), and intestinal acetone powders from rat (I1630) were purchased from Sigma-Aldrich (Steinheim, Germany). Ammonium molybdate (AT0330-5) was bought from Tecsiquim (Mexico City, Mexico).

### 4.4. HPLC Analysis

The HPLC profiles were obtained using a 1260 HPLC instrument (Agilent, San Jose, CA, USA) equipped with a G1311B Quaternary Pump, a G1367E Autosampler and an Agilent G1315C UV diode array detector (DAD). System control, data collection, and data processing were accomplished using OpenLAB LC 1260 chromatography software. Elution was carried out at a flow rate of 0.35 mL/min with water as solvent A containing 0.1% formic acid and acetonitrile (MeCN) as solvent B, the elution gradient was carried out by starting with a mixture of 99:1 (A:B), increasing the amount of solvent B as follows: 75:25 (A:B) at 14 min, 70:30 (A:B) at 14–18 min, 65:35 (A:B) at 18–22 min, 5:95 (A:B) at 22–27 holding this mixture for a min and 99:1 (A:B) at 28–30. The separation was carried out using a Luna Omega Polar C18, 50 × 2.1 mm of internal diameter., 1.6 μm) reverse phase column (Phenomenex, San Jose, CA, USA). The column temperature was kept at 35 °C. Working solutions of samples (extracts and standards) were prepared by dissolving 10 mg and 15 mg of *E. cymosum* and *A. firma* extracts, respectively, with the appropriate solvent for each extract (1mL of H2O for *A. firma* and 2 mL of a mixture of H2O:MeCN:MeOH; 50:25:25 for *E. longifolium*) and 2 mg of standard in 5 mL of methanol (MeOH) and diluted to 100 μg/mL. All samples were filtered on membrane filters (PTFE, 0.20 μm) and injected (3 μL). For UV detection, the wavelength program was set at an acquisition of λ 240, 254, 280, 320 and 365 nm; the UV spectra were recorded from 230 at 400 for *E. longifolium* profile and from 180 at 390 nm for *A. firma*. The identification of caffeic acid (**1**), chlorogenic acid (**2**) and rosmarinic acid (**3**) in *E. longifolium* was carried out as previously described [33].

### 4.5. Experimental Animals

For the in vivo hypoglycemic tests, ninety-nine 8-week-old Wistar rats were used, while twenty 8-week-old BALB/c mice were acquired for the acute toxicity tests. All animals were obtained from the bioterium of the School of Sciences, UNAM, Mexico City, Mexico and maintained with free access to food and water under standard conditions (25 °C, 55% humidity and 12 h light:12 h dark periods). All methods applied to the animals in this study were approved by the Academic Ethics and Scientific Responsibility Commission (CEARC) of the School of Sciences, UNAM, Mexico City, Mexico (P_2021_05_01) and carried out according to the Guide for the Care and Use of Laboratory Animals, Washington DC, USA [39].

### 4.6. Acute Oral Toxicity Tests

To test the acute safety of the plant extracts, acute toxicity tests were performed by orally giving a maximum single dose of 2000 mg/kg b.w. to five BALB/c mice for each extract, according to the OECD guidelines 425 [17]. First, one mouse was administered with the tested extract and closely observed for 30 min to detect any behavioral or physical abnormality. Next, it was observed every four hours for 24 h and then frequently for 14 days. Based on the outcomes, the maximum single dose was given to the remaining four mice to repeat the procedure previously described.

### 4.7. Induction of Hyperglycemia

The STZ-NA hyperglycemic model [40] was selected to test the hypoglycemic effect of the plant extracts. In brief, Wistar rats were fasted for 12 h and then administered intraperitoneally with 150 mg/kg b.w. of a fresh NA solution prepared with physiological solution. Fifteen min later, they were injected intravenously with 65 mg/kg b.w. of a STZ solution prepared one day before in 0.1 M acetate buffer, pH 4.5. Animals with non-fasting blood glucose above 180 mg/dL were selected one week after the induction to perform the experiments.

### 4.8. Assessment of Hypoglycemic Effect

The animals were divided into 11 groups with nine individuals each: the normoglycemic control (1) and the negative hyperglycemic control (2) were administered with physiological solution; the positive hyperglycemic control (3) was given the drug glibenclamide (Euglucon^®^, 5 mg/kg b.w.); two hyperglycemic groups were administered with *A. firma* rhizome aqueous extract, one with the traditional dose (4) and another with the traditional high dose (5) (16 mg/kg b.w. and 160 mg/kg b.w., respectively); two hyperglycemic groups were given *A. firma* rhizome EtOH extract, one with the traditional dose (6) and another with the traditional high dose (7) (37 mg/kg b.w. and 374 mg/kg b.w., respectively); two hyperglycemic groups were administered with *E. longifolium* aerial part aqueous extract, one with the traditional dose (8) and another with the traditional high dose (9) (30 mg/kg b.w. and 310 mg/kg b.w., respectively); and two hyperglycemic groups were given *E. longifolium* aerial part EtOH extract, one with the traditional dose (10) and another with the traditional high dose (11) (32 mg/kg b.w. and 318 mg/kg b.w., respectively). Both glibenclamide and extracts were dissolved in physiological solution.

Blood glucose levels were measured at baseline and, after the treatment administration by gavage, monitored every hour for 3 h [41]. Samples were obtained from the tail vein and quantified in duplicate in using glucometers (Accutrend^®^ Plus, Roche Diagnostics International AG, Rotkreuz, Switzerland).

### 4.9. Glucose 6-phosphatase Inhibition Assay

The activity of G6Pase enzyme was assessed by a colorimetric test which detects the blue phosphomolybdate formation [42,43]. Microsomal fractions obtained from livers of Wistar rats were resuspended in buffer (40 mM imidazole, 250 mM sucrose, pH 7) and mixed with extracts or chlorogenic acid (control) at concentrations ranging from 2 μg/mL to 5000 μg/mL. The reaction was started with the addition of 20 mM glucose 6-phosphate and incubated at 20 °C for 20 min. At the end of incubation, 900 μL of stop solution (0.42% ammonium molybdate in 1 N H_2_SO_4_, 10% SDS, and 10% ascorbic acid) was added to the reaction mixture and incubated at 45 °C for 20 min. The absorbances of reaction mixtures were obtained at 830 nm. Two independent experiments in triplicate were carried out.

### 4.10. Fructose 1,6-bisphosphatase Inhibition Assay

To measure the inhibition of FBPase activity, a previous procedure was followed [33,44]. Before starting the assay, the color reagent was prepared by adding 0.12% malachite green in 5 volumes of water and 1 volume of H_2_SO_4_. On the day of the assay, 1 volume of 7.5% molybdate the ammonium was mixed with 4 volumes of the color reagent. Then, 0.17% of TWEEN 20 was added. The reaction assay was prepared by adding buffer (5 μM EDTA, 5 mM MgCl_2_, 50 mM Tris-HCl, pH 7.2) enriched with 0.1 mM fructose 1,6-bisphosphate and inhibitory samples (AMP or extract) at concentrations ranging from 2 μg/mL to 5000 μg/mL. Cytosolic supernatant (diluted 1:10) obtained from livers of Wistar rats was used to start the reaction, and then it was incubated for 15 min at 20 °C. After that, color reagent was added, and a second incubation was made for 10 min at 20 °C. The absorbances were obtained at 630 nm. Two independent experiments were performed in triplicate.

### 4.11. α-glucosidase Inhibition Assay

A modified method of a previously performed α-glucosidase assay was used [33,45]. First, in 100-μL reaction volumes, 0.1 M phosphate buffer at pH 6.8, inhibitor samples (acarbose (Aurax^®^, Mexico City, Mexico) or extracts) at concentrations ranging from 200 μg/mL to 2000 μg/mL, and an α-glucosidase solution prepared from rat intestinal acetone powder were placed and incubated at 35 °C for 3 min. Afterwards, 2 mM 4-nitrophenyl α-D-glucopyranoside (p-NPG) was added to start the enzymatic activity and, subsequently, the reaction was incubated at 35 °C for 30 min. Finally, the absorbances were determined at 405 nm. Two independent experiments in triplicate were performed.

### 4.12. Statistical Analysis

Data were represented as mean ± SEM. For the 3-h acute tests, data were assessed for normal distribution and log transformed as necessary. Then, ordinary one-way ANOVA and Tukey’s post-hoc tests were carried out to compare the means among groups in each time, while repeated measures ANOVA and Dunnet’s post-hoc tests were performed to compare the means with their baseline. The respective non-parametric tests were applied if normality was not obtained, even after log transformation. *P*-values less than 0.05 were considered significant. Additionally, the areas under the curve (AUC) of blood glucose were calculated and compared using ordinary one-way ANOVA and Tukey’s post-hoc tests.

To obtain the IC_50_ values, absorbances were transformed to activity percentage as follows:

Enzyme activity (%) = (A_S_ − A_SB_)/(A_C_ − A_CB_) × 100 (1)

where, A_S_ is the absorbance of the inhibitor sample at a specific concentration, A_SB_ is the blank of the inhibitor sample, A_C_ is the highest absorbance (without inhibitor), and A_CB_ is the blank of the highest absorbance. Then, percentage values were plotted on concentration-response curves to find the best fitting non-lineal regression model (three or four parameters).

## 5. Conclusions

The traditional therapeutic use of the aerial part of *E. longifolium* and the rhizome of *A. firma* as hypoglycemic agents was confirmed in a hyperglycemic animal model. Furthermore, both plants inhibited two key enzymes involved in the liver glucose output, which mainly controls glucose levels in the fasting state. For *E. longifolium,* the presence of rosmarinic and chlorogenic acids could explain the bioactivity. Further studies are needed to discard other action mechanisms that can act in a synergistic way to produce the observed hypoglycemic effect.

## Figures and Tables

**Table 1:** Blood glucose values from 3-h acute tests expressed as mg/dL (mean ± SEM, *n* = 9).

Group		Dose	0 min	60 min	120 min	180 min
Normoglycemic Control		n/a	120 ± 3 ^a^	118 ± 2 ^a^	116 ± 2 ^a^	119 ± 3 ^a^
Hyperglycemic Control		n/a	192 ± 4	190 ± 5 ^b^	188 ± 6 ^b^	193 ± 5 ^b^
Hyperglycemic + Glibenclamide Control		5 mg/kg	187 ± 5	152 ± 6 ^a,^*	128 ± 3 ^a,^*	124 ± 3 ^a,^*
Hyperglycemic + *A. firma* Rhizome	Aqueous Extract	16 mg/kg	195 ± 4	157 ± 5 ^a,^*	136 ± 4 ^a,^*	132 ± 3 ^a,^*
		160 mg/kg	190 ± 3	159 ± 5 *	150 ± 3 ^a,b,^*	140 ± 2 ^a,^*
	EtOH Extract	37 mg/kg	199 ± 5	166 ± 4 *	149 ± 4 ^a,b,^*	147 ± 5 ^a,b,^*
		374 mg/kg	205 ± 4	184 ± 5 ^b^	158 ± ^a,b,^*	153 ± 4 ^a,b,^*
Hyperglycemic + *E. longifolium* Aerial Part	Aqueous Extract	30 mg/kg	197 ± 6	169 ± 7 *	148 ± 4 ^a,b,^*	149 ± 4 ^a,,b^*
		310 mg/kg	204 ± 7	164 ± 7 ^a,^*	135 ± 4 ^a,^*	136 ± 5 ^a,^*
	EtOH Extract	32 mg/kg	186 ± 6	156 ± 5 ^a,^*	141 ± 3 ^a,^*	121 ± 3 ^a,^*
		318 mg/kg	193 ± 5	152 ± 4 ^a,^*	141 ± 3 ^a,^*	132 ± 3 ^a,^*

Rows: *—statistically significant difference versus its initial time at *p* < 0.05. Columns: ^a^—indicates statistically significant difference versus hyperglycemic group in that time at *p* < 0.05; ^b^—indicates statistically significant difference versus glibenclamide control in that time at *p* < 0.05.

## Data Availability

Data are available upon request.
